# Diagnostic Potential of Exosomal and Non-Exosomal Biomarkers in Lung Cancer: A Comparative Analysis Using a Rat Model of Lung Carcinogenesis

**DOI:** 10.3390/ncrna11030047

**Published:** 2025-06-16

**Authors:** Sherien M. El-Daly, Sahar S. Abdelrahman, Amira Mohamed Abd El-Jawad, Mahmoud A. Abdel-Monem, Gamila S. M. El-Saeed

**Affiliations:** 1 Medical Biochemistry Department, Medical Research and Clinical Studies Institute, National Research Centre, Dokki, Giza 12622, Egypt; 2 Cancer Biology and Genetics Laboratory, Centre of Excellence for Advanced Sciences, National Research Centre, Giza 12622, Egypt; 3 Department of Pathology, Faculty of Veterinary Medicine, Cairo University, Giza 12211, Egypt

**Keywords:** extracellular vesicles, exosomes, liquid biopsy, lung cancer, miRNAs, tumor marker, diagnosis

## Abstract

Background: Identifying liquid biopsy biomarkers with high efficacy is crucial for cancer diagnosis. Exosomal cargo, including miRNAs and proteins, offers enhanced stability in biofluids compared with their free circulating forms, but direct comparisons of their diagnostic performance remain limited. This study evaluates and compares the diagnostic value of selected miRNAs and protein markers in exosomal versus non-exosomal fractions across stages of lung carcinogenesis in a rat model. Methods: Lung cancer was induced in rats, and blood and lung tissue samples were collected at consecutive stages of tumor induction. We investigated the expression patterns of key miRNAs (miR-19b, miR-21, and miR-145) in exosomes, serum, and tissue and quantified levels of tumor biomarkers CEA and CYFRA 21-1 in exosomal and serum fractions. Results: Our results revealed distinct expression patterns of the evaluated miRNAs across exosomes, serum, and tissue, throughout different stages of tumor induction. The expression of exosomal miRNAs dynamically changed in parallel with the tumor induction process, demonstrating high diagnostic efficacy. Specifically, exosomal miR-19b and miR-21 were significantly upregulated from an early induction stage, whereas their serum and tissue forms increased only during the late stages of induction. On the other hand, miR-145 was consistently downregulated across all fractions at every stage. Both exosomal and serum CEA levels increased significantly during tumor induction, while serum CYFRA 21-1 outperformed its exosomal counterpart. Strong positive correlations linked exosomal miR-19b and miR-145 with their non-exosomal counterparts, while moderate correlations were seen for miR-21 and the protein markers. Conclusions: Our findings underscore the value of integrating exosomal biomarkers in liquid biopsies, highlighting their potential to improve early detection and monitoring of lung cancer development.

## 1. Introduction

Lung cancer is a major cause of cancer-related deaths globally, affecting both males and females. Patients with lung cancer frequently experience disease progression, which includes poor clinical outcomes, complications, and recurrences. Many cases of lung cancer are diagnosed at an advanced stage, with over 70% of patients presenting with advanced or metastatic disease at the time of diagnosis [1,2]. Therefore, early diagnosis is crucial for efficient disease management and preventing the progression of cancer. Individuals with lung cancer often depend on small tissue biopsies for diagnosis. However, with tumor lesions not being readily visible, the ease of access to the central tumor and repeated follow-up would be restricted [3]. Hence, there is an urgent need for a well-established liquid biopsy protocol to address this clinical requirement.

MicroRNAs (miRNAs) have emerged as highly appealing diagnostic tools due to their stability and reliability. These small-sequence non-coding RNAs play vital roles in post-transcriptional gene regulation and have been linked to various biological processes, including carcinogenesis [4]. The stability of miRNAs in biofluids, along with their tissue-specific expression patterns, makes them promising candidates for non-invasive diagnostic tests [5]. However, circulating miRNAs are not naturally resistant to endogenous RNases and require protection from degrading enzymes [6]. miRNAs can be detected in the circulation associated with protein complexes or incorporated in extracellular vesicles (EVs). All of these carriers appear to have a unique miRNA profile. Circulating miRNAs have been identified in a highly stable form within EVs protected from endogenous RNase activity [7].

Exosomes are bilayer lipid-membrane vesicles with a 30–160 nm size range. They are a class of extracellular vesicles of endosomal origin released by all cells under different conditions to mediate intercellular communication by transferring their encapsulated cargo (components) to other cells. Exosome incorporates proteins, lipids, metabolites, and nucleic acids that mirror their cell of origin and its physiological and biological state [8]. Hence, these contents could be utilized as a source of biomarkers. A plethora of reports suggest that circulating exosomes hold promise as biomarkers, given that they represent the phenotypic condition of the parent cell, they can be collected using a non-invasive procedure, and their content is protected from degradation [9,10,11].

Exosomal components, such as miRNAs and proteins, could have several advantages over their non-exosomal counterparts, such as their enhanced stability in circulation [12]. However, a complete understanding of the diagnostic value of exosomal biomarkers relative to non-exosomal biomarkers is still being delineated, especially in lung cancer. Some studies have supported the superiority of exosomal miRNAs over non-exosomal cell-free miRNAs as promising biomarkers [12,13,14]. Few studies have investigated which compartment exhibits more accuracy, sensitivity, and specificity. Therefore, the main objective of this study is to assess the diagnostic potential and compare the sensitivity and specificity of selected miRNAs and protein tumor markers in exosomal versus non-exosomal fractions, as diagnostic tools for accurately reflecting the development of lung cancer. To characterize the aberrant expression patterns of these biomarkers during lung carcinogenesis, lung cancer was experimentally induced in a rat model, and blood and lung tissue samples were collected at defined stages throughout tumor development. We focused our study on a carefully selected panel of miRNAs with well-documented and contrasting roles in cancer pathogenesis, oncogenic versus tumor suppressor. Our selection includes two established oncogenic miRNAs, miR-21 and miR-19b, and a prominent tumor suppressor miRNA, miR-145. miR-21 is a universally recognized oncomiR, consistently found to be significantly upregulated in various human cancers, including both non-small cell lung cancer (NSCLC) and small cell lung cancer (SCLC) [15]. It exerts its pro-tumorigenic effects primarily by targeting key tumor suppressor genes such as PTEN and PDCD4, thereby promoting cell proliferation, survival, invasion, and inhibiting apoptosis [16]. Similarly, miR-19b is a potent oncogenic miRNA and a core member of the highly studied miR-17-92 cluster, which is frequently amplified and overexpressed in lung cancers. miR-19b promotes cell survival and proliferation by repressing pro-apoptotic factors, such as BIM (BCL2L11), and targeting PTEN [17]. This action functionally overlaps with the role of miR-21. In contrast, miR-145 functions predominantly as a tumor suppressor miRNA, commonly observed to be downregulated in lung cancer compared to normal tissue. It acts by inhibiting crucial oncogenes, including MYC, NRAS, and OCT4, thereby suppressing cell proliferation, migration, and invasion [18,19]. The careful selection of this panel with their contributions to critical cancer pathways enables a more comprehensive investigation of the dynamic miRNA landscape during tumor induction. This approach is more effective than studying a single miRNA. Furthermore, all three miRNAs have been previously detected in circulating biofluids, suggesting their potential accessibility as diagnostic biomarkers [15,20]. Concurrently, serum-derived exosomes and their corresponding serum samples were also analyzed for the widely recognized protein tumor markers Carcinoembryonic Antigen (CEA) and Cytokeratin Fragment Antigen-21 (CYFRA 21-1). These markers are clinically utilized as diagnostic, prognostic, and monitoring biomarkers for lung cancer [21]. This comparative study aims to comprehensively assess the expression dynamics of selected miRNAs and protein tumor biomarkers in exosomal and non-exosomal fractions (serum and tissue), providing valuable insights into the potential use of exosomal markers for lung cancer diagnosis. Our findings revealed distinct expression patterns across these biological fractions, suggesting a complex interplay in biomarker dynamics during lung carcinogenesis.

## 2. Results

### 2.1. Histopathological Evaluation

**Figure 1:**
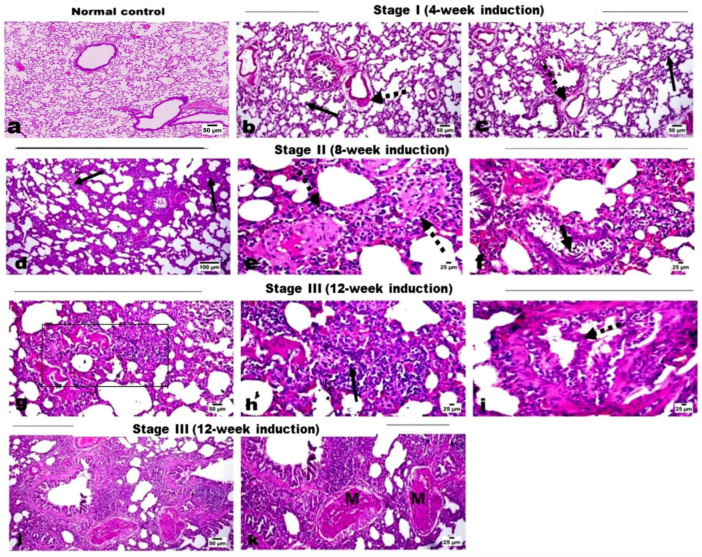
H&E-stained photomicrographs for lung tissue sections. (**a**) Normal control group showing normal alveoli and interalveolar septa. (**b**,**c**) Stage I group showing mild thickening of the interalveolar septa (arrow) and edema in the pulmonary blood vessels’ walls (dashed arrow). (**d**–**f**) Stage II rats showing thickening of the interalveolar walls (arrow) with infiltration of mononuclear inflammatory cells and eosinophils, and also in the perivascular area (dashed arrow), and desquamated bronchial epithelium (short arrow). (**g**–**k**) Stage III rats showing alveolar collapse (area) with marked infiltration of inflammatory cells and hyperplastic alveolar epithelium, which has scattered mitosis (arrow), polypoid hyperplasia of the bronchiolar epithelium (dashed arrow), lung consolidation, and vascular intimal proliferation with medial hyperplasia (M).

**Figure 2:**
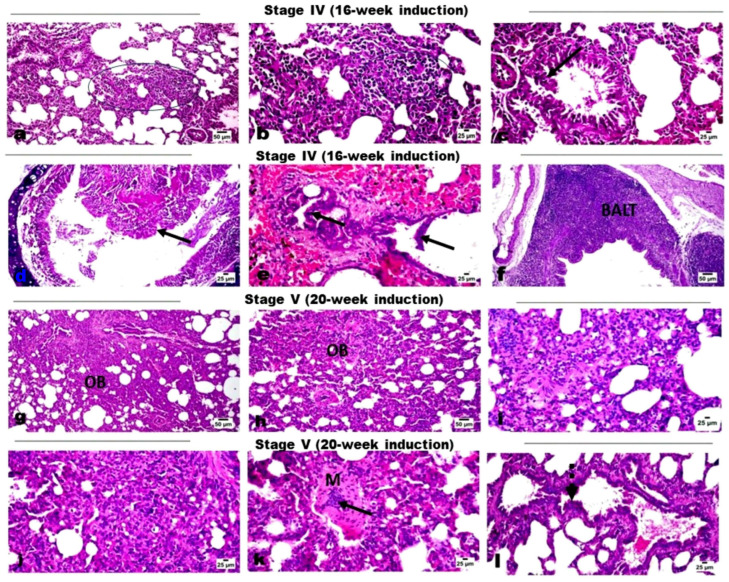
H&E-stained photomicrographs for lung tissue sections. (**a**–**f**) Stage IV rats showing multifocal areas of alveolar lining hyperplasia (square) with increased cellular atypia, polypoid hyperplasia of the bronchial epithelium (arrow), hyperplasia of the peri-bronchial lymphoid follicles (BALT). (**g**–**l**) Stage V rats showing multifocal alveolar atypical dysplasia with complete obliteration of the alveolar lumen (OB), solid adenocarcinoma (**i**,**j**), marked vascular intimal proliferation obliterating the vessels’ lumen (arrow) with medial hyperplasia (M), and focal metaplastic changes (dotted arrow) of the bronchial epithelium.

Microscopic examination of lung tissues of the control rats showed normal histological structure without any histological alterations (Figure 1a). Examination of lung tissues from groups that received DEN along with PB administration showed marked histopathological changes across induction stages as detailed. Lung tissue sections of the Stage I group (4-week induction) revealed edema in pulmonary blood vessels’ walls with mild thickening of the interalveolar septa (Figure 1b), as well as a mild proliferation of the bronchiolar epithelial linings and few scattered infiltration of inflammatory cells (Figure 1c). Lung tissue of the Stage II group (8-week induction) revealed thickening of the interalveolar walls with proliferation of alveolar epithelium and infiltration of mononuclear inflammatory cells (Figure 1d) mixed with some eosinophiles, particularly in the perivascular area (Figure 1e). The proliferated cells obliterated the normal alveolar space and showed multifocal alveolar collapse. The bronchiolar epithelium showed degenerative changes, marked desquamation into the lumen, and focal to diffuse hyperplastic changes (Figure 1f). The nuclei of the hyperplastic cells were vesicular and showed focal stratification. Regarding lung tissue sections of the Stage III group (12-week induction), serious mass infiltration of inflammatory cells was observed, along with hyperplastic alveolar epithelium obliterating the normal alveolar lumen, resulting in alveolar collapse (Figure 1g). The hyperplastic cells showed scattered mitosis (Figure 1h). Marked hyperplasia of the bronchiolar epithelium was evident with polyps’ formation into the lumen; the hyperplastic cells exhibited vesicular and anisokaryotic nuclei (Figure 1i). Some sections showed marked vascular intimal proliferation with medial hyperplasia (Figure 1j). Foci of lung consolidation was observed as a result of alveolar hyperplasia, where the hyperplastic cells showed some degree of cellular atypia (Figure 1k). Tissue sections of the Stage IV group (16-week induction) revealed several focal areas of alveolar hyperplasia, with increased cellular atypia completely obliterating the alveolar lumen (Figure 2a,b). The alveolar structure was absent in those areas and replaced by proliferating cells. The bronchiolar epithelium showed marked atypic hyperplastic changes (Figure 2c), with multifocal hyperplasia and stratification (Figure 2d) and polypoid appearance obliterating the bronchiolar lumen (Figure 2e), as well as marked hyperplasia of the peri-bronchial lymphoid follicles (Figure 2f). The Stage V group (20-week induction) revealed multifocal alveolar atypical dysplasia, with complete obliteration of the alveolar lumen (Figure 2g,h) where the alveoli showed dysplastic proliferated cells with moderate anisokaryosis, anisocytosis, and frequent mitosis. Dysplastic cells formed solid sheets with marked cellular atypia and frequent mitosis, consistent with solid adenocarcinoma (Figure 2i,j), and also showed attempts at forming a lepidic pattern in some areas. Most of the pulmonary vessels showed marked intimal proliferation, which sometimes appeared to obliterate the vessel’s lumen with obvious medial hyperplasia (Figure 2k). Focal metaplastic changes of the bronchial epithelium were evident in these rats (Figure 2l).

### 2.2. Characterization of Isolated Exosomes

**Figure 3:**
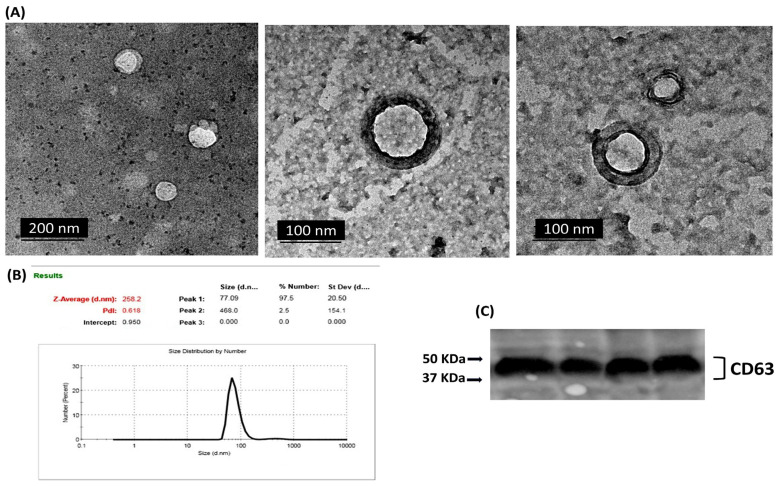
Exosomes isolated from serum samples were characterized by (**A**) transmission electron microscopy (TEM), where cup-shaped vesicle structures were identified as exosomes. (**B**) dynamic light scattering (DLS), where particle sizes were normally distributed with a mean value of approximately 77 nm. Size (d.nm) = Size in diameter nanometers, St Dev (d.nm) = Standard Deviation in diameter nanometers. (**C**) Western blot analysis for exosomal surface marker CD63 in a set of isolated exosome samples.

Transmission electron microscopy (TEM) analysis for serum-derived exosomes revealed the presence of particles with vesicular morphologies. Round or cup-shaped vesicles were observed with heterogeneous sizes ranging from approximately 40 to 140 nm, characteristic of exosomes (Figure 3A). The size and diameter of extracted exosomes were further analyzed using dynamic light scattering (DLS). The results revealed that the particle sizes were normally distributed with a mean value of approximately 77 nm (Figure 3B). The exosomal marker protein CD63 was observed in the exosomal protein lysates using Western blot analysis (Figure 3C). These results confirm that the vesicles extracted from serum samples display typical exosomal characteristics.

### 2.3. Expression Patterns of miRNAs in Exosomal and Non-Exosomal Fractions

**Figure 4:**
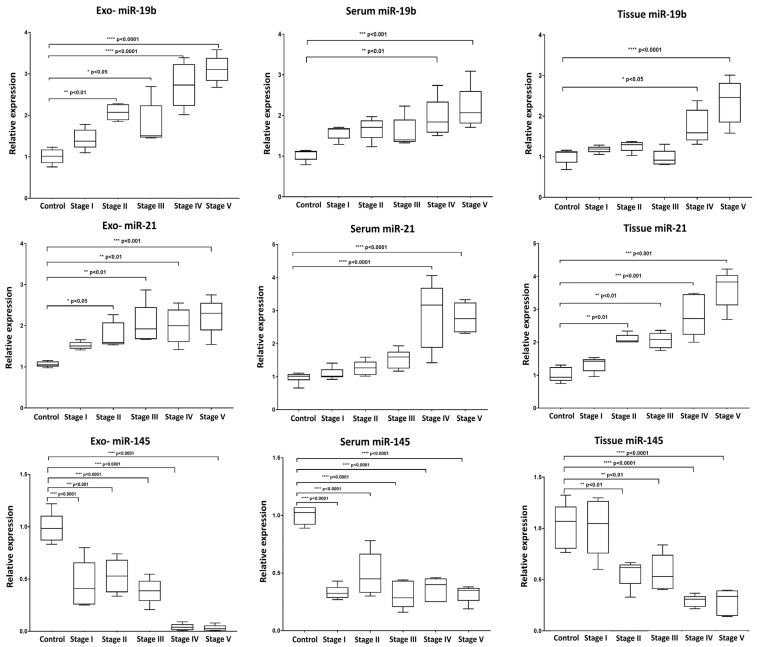
Box plot whiskers representing the relative expression levels of selected miRNAs (miR-19b, miR-21, and miR-145) in extracted exosome, serum, and tissue samples at each time point of collection. Statistical significance between groups was assessed using one-way ANOVA followed by Tukey’s post hoc multiple comparison test. * *p* < 0.05; ** *p* < 0.01; *** *p* < 0.001; **** *p* < 0.0001.

Our analysis revealed a distinct expression pattern for the selected miRNAs between the various fractions (exosomes, serum, and tissue) and across the different stages of induction (Figure 4). For miR-19b, Exo-miR-19b exhibited a dynamic upregulation during the induction stages, with statistically significant alterations observed starting from Stage II (8-week induction) with the initial development of hyperplastic changes. In contrast, serum miR-19b and tissue miR-19b exhibited significantly elevated expression only in the last two stages of induction, associated with dysplasia and carcinoma development. For miR-21, the expression pattern of either the exosomal or tissue miR-21 exhibited dynamic upregulation throughout the various induction stages, with significant expression starting from Stage II (8-week induction). On the other hand, compared to the control, the significant overexpression of serum miR-21 was only detected in the late stages of induction (Stage IV and Stage V), associated with dysplasia and carcinoma development, with no significant alterations during the early induction cycles. For miR-145, a comparable pattern of dynamic downregulation was detected across all three forms of miR-145 (exosomal, serum, and tissue) throughout the tumor induction stages. The expression levels of miR-145 were significantly reduced at each induction stage compared to the control, indicating an almost consistent response across exosomal and non-exosomal fractions.

### 2.4. Levels of CEA and CYPF21 in Serum and Exosomes

**Figure 5:**
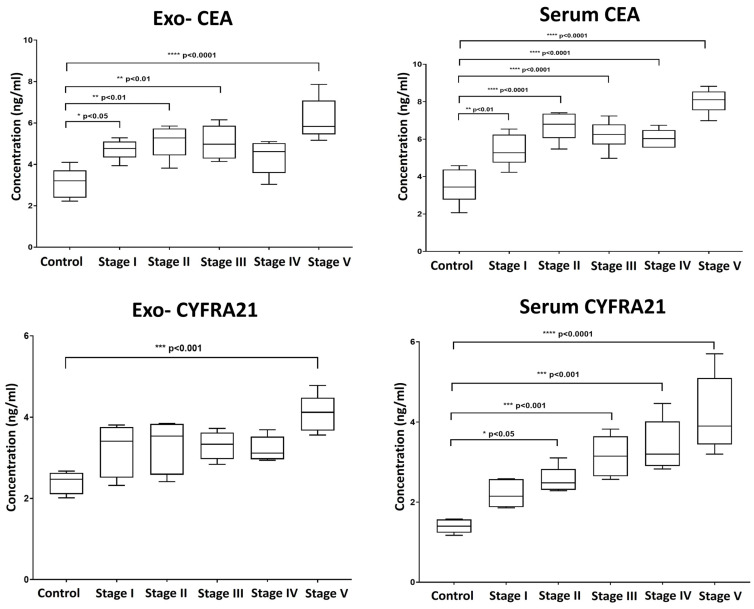
Box plot whiskers representing the mean concentration values (ng/mL) for Carcinoembryonic Antigen (CEA) and Cytokeratin Fragment Antigen-21 (CYFRA 21-1) in exosomal and matched serum samples at each time point of collection. Significance was estimated between groups using one-way ANOVA, followed by Tukey’s post hoc multiple comparison test. * *p* > 0.05; ** *p* > 0.01; *** *p* > 0.001; **** *p* > 0.0001.

We also evaluated the levels of the established clinical tumor biomarkers Carcinoembryonic Antigen (CEA) and Cytokeratin Fragment Antigen-21 (CYFRA 21-1) in serum-derived exosomes and corresponding serum samples (Figure 5). Our findings revealed that the pattern of elevation in the level of CEA was almost similar in both extracted exosomes and serum, with dynamic overexpression observed throughout the progressive stages of tumor induction, reaching high statistically significant levels with the development of carcinoma.

In contrast, serum levels of CYFRA 21-1 were more indicative, showing a consistent increase aligned with the stages of induction compared to exosomal CYFRA 21-1. Exosomal CYFRA 21-1 levels only exhibited a significant rise at the final stage of induction, with no significant changes detected during earlier stages (Figure 5).

### 2.5. Diagnostic Performance of Exosomal and Non-Exosomal miRNAs and Protein Tumor Markers

We conducted receiver operating characteristic (ROC) curve analysis to evaluate the diagnostic performance of our selected miRNAs (miR-19b, miR-21, and miR-145) and the protein tumor biomarkers CEA and CYFRA 21-1. We compared the results with those from matched non-exosomal fractions. As we focus on early diagnosis, we conducted the ROC curve analysis on the group representing the first induction stage (Table 1, Supplementary Figure S1).

The ROC curve analysis revealed that our selected panel of miRNAs demonstrated good diagnostic accuracy for lung carcinogenesis (Table 1, Supplementary Figure S1). However, the performance varied depending on the miRNA and the analyzed fraction (exosome, serum, or tissue). For miR-19b, the serum form exhibited the highest diagnostic accuracy, with an area under the ROC curve (AUC) equal to 1.000 and 100% sensitivity and specificity. Exosomal miR-19b also exhibited excellent performance with an AUC of 0.920, 80% sensitivity, and 100% specificity, followed by tissue miR-19b with an AUC of 0.880, 80% sensitivity, and 100% specificity. On the other hand, for miR-21, the exosomal fraction demonstrated the highest diagnostic accuracy compared to its serum and tissue counterparts, with an AUC of 1.000 and 100% sensitivity and specificity. Tissue miR-21 followed with an AUC of 0.880 and 80% sensitivity and specificity, while serum miR-21 showed an AUC of 0.625, with lower sensitivity and specificity (66.7%). For miR-145, interestingly, both the exosomal and serum forms showed excellent diagnostic performance, each achieving an AUC of 1.000 with 100% sensitivity and specificity. In contrast, tissue miR-145 showed lower diagnostic power with an AUC of 0.520 and 60.0% sensitivity and specificity.

Regarding the conventional protein tumor biomarkers CEA and CYFRA 21-1, both exosomal and serum CEA exhibited comparable and high diagnostic performance (AUC = 0.960). However, exosomal CYFRA 21-1 showed a slightly lower diagnostic performance (AUC = 0.880) compared to serum CYFRA 21-1 (AUC = 1.000), indicating that the serum form of CYFRA 21-1 could be more accurate for diagnosis.

### 2.6. Correlation Between Exosomal and Non-Exosomal Biomarkers

**Figure 6:**
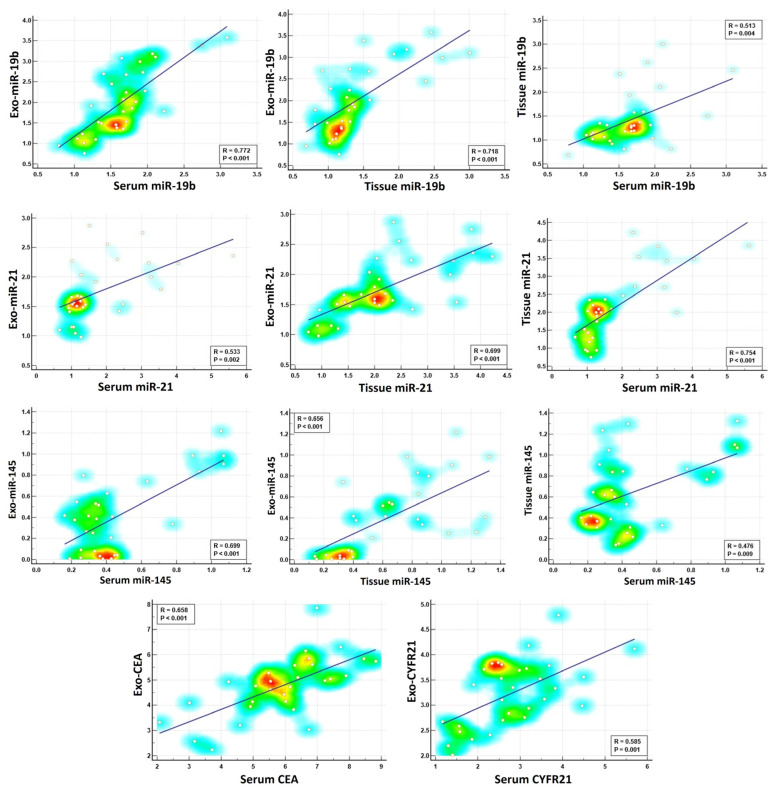
Spearman’s correlation analysis of selected miRNAs and protein tumor markers across exosomal, serum, and tissue fractions. The color intensity represents the density of data points.

In our study, we were interested in investigating the potential connections between exosomal and non-exosomal contents to evaluate if they are influenced by similar biological processes. We performed Spearman’s correlation analysis, comparing each biomarker in its exosomal form with its corresponding serum or tissue counterpart. Although the data revealed a significant correlation between exosomal miRNAs and their corresponding non-exosomal forms, and the same was applied for protein tumor biomarkers, the correlation varied in strength depending on the measured parameter (Table 2, Figure 6). For example, we identified strong positive correlations for exosomal miR-19b with their serum or tissue counterparts (r = 0.772 ** and r = 0.718 **, respectively), suggesting a coordinated elevated response during lung carcinogenesis. On the other hand, moderate positive correlations were detected for miR-21 and miR-145 between their exosomal and non-exosomal fractions, indicating a potential association but with varying degrees of synchronicity. Similar to the miRNA data, the correlations between exosomal and serum levels of the protein tumor markers (CEA and CYFRA21) varied in strength. We found that the correlation between exosomal and serum levels of CEA was higher (r = 0.658 **) than that for exosomal and serum levels of CYFRA21 (r = 0.585 **), suggesting that both exosomal and serum CEA levels are positively connected and are elevated in parallel during lung carcinogenesis. The correlation analysis also revealed a connection between exosomal markers. Specifically, Exo-miR-19b exhibited a strong correlation either positively with Exo-miR-21 (r = 0.676 **) or negatively with Exo-miR-145 (r = −0.790 **). For exosomal protein tumor markers, a positive correlation was detected between CEA and CYFRA21 (r = 0.568 **). Moreover, a negative correlation was detected between these exosomal protein markers and Exo-miR-145, with correlation values of r = −0.565 ** and −0.668 **, respectively (Table 2). Overall, the correlation data suggest that exosomal miR-19b and miR-145 might be promising candidates for early lung cancer detection due to their strong correlation with their non-exosomal fractions.

## 3. Discussion

Cancer diagnosis at an early stage is a key factor in extending patients’ lives and lowering cancer-related mortality. Genetic information from a single tissue biopsy is restricted and does not capture tumor heterogeneity. Therefore, liquid biopsies have emerged as promising monitoring tools that could provide a comprehensive genetic profile of malignant tumors and can be repeated to track tumor progression, allowing for dynamic surveillance of genomic changes [3].

Exosomes have emerged as one of the most promising biomarkers in liquid biopsy. Exosomes carry a stable cargo of nucleic acids, lipids, metabolites, and proteins reflective of their cell of origin, making them a reliable source of diagnostic/prognostic biomarkers. The encapsulation of these molecules within the lipid bilayer of exosomes protects them from degradation by extracellular RNases and proteases, ensuring their stability during circulation in bodily fluids. This stability allows for the reliable identification and quantification of exosomal biomarkers [10]. Moreover, the selective packaging of miRNAs and proteins into exosomes enhances their diagnostic value, as they serve as a snapshot of cellular activity during cancer progression [22].

The potential utility of exosomal cargo, such as miRNAs and proteins, as diagnostic biomarkers for lung cancer needs further elucidation compared to their non-exosomal counterparts. Our study aimed to comprehensively assess the diagnostic potential of exosomal biomarkers as diagnostic biomarkers for lung carcinogenesis compared with their non-exosomal counterparts.

To the best of our knowledge, this is the first study to comprehensively evaluate and compare the diagnostic potential of exosomal miRNAs and protein tumor biomarkers with their non-exosomal counterparts across stages of lung cancer development.

Our selection of exosomal biomarkers includes three miRNAs (miR-19b, miR-21, miR-145), previously identified as potential diagnostic biomarkers and two clinically applied conventional protein tumor biomarkers, CEA and CYFRA21. We opted to investigate the expression of miR-19b, miR-21, and miR-145 based on their well-documented roles in lung cancer pathogenesis and their potential use as diagnostic/prognostic biomarkers in various types of cancer [15,23].

miR-19b is a member of the miR-17-92 oncogenic cluster and was reported to be involved in lung cancer development by inhibiting apoptosis through phosphatase and tensin homolog (PTEN) and TP53 [24]. miR-19b is identified as a promising diagnostic marker for NSCLC. miR-19b expression was reported to be elevated in both tumor tissue and the circulation of lung cancer patients, and this elevated expression in both often correlates with more aggressive disease and poorer outcomes. Both our findings and previous studies indicate that elevated expression of miR-19b is associated with poor prognosis [23,25]. The exosomal form of miR-19b was also reported to be a promising diagnostic marker for pancreatic cancer, with a diagnostic power superior to that of serum CA19-9 in differentiating cancer patients from healthy volunteers [26].

miR-21 is one of the most established oncogenic miRNAs frequently overexpressed in various types of cancer, including NSCLC [27,28]. It exerts its function by targeting and downregulating the expression of essential tumor-suppressive genes such as PTEN and the suppressor of cytokine signaling 1 and 6 (SOCS1/6), thereby disrupting apoptotic pathways and promoting aberrant cell growth and proliferation in NSCLC [16,29]

miR-21 is also extensively investigated as a promising diagnostic biomarker [30]. The increased serum levels of miR-21 have been associated with advanced TNM stages and distant metastasis in NSCLC [15,31]. All forms of miR-21, either as exosomal, serum/plasma-derived, or tissue-derived, have been reported as promising biomarkers for diagnosis and prognosis, and even as predictive biomarkers [32,33]. A meta-analysis encompassing ten different cancer types suggests that exosomal miR-21 may serve as a universal fluid-based biomarker for cancer. Compared to conventional markers, such as CA19-9 and CEA, exosomal miR-21 appears to offer superior diagnostic accuracy [34].

In contrast to miR-19b and miR-21, miR-145 has been reported to be downregulated in many types of cancer, including lung cancer. It has been described as a tumor suppressor miRNA involved in regulating cell differentiation and proliferation [35,36,37,38]. The miR-145 gene is located at the 5p32 chromosomal region, the expression of which is regulated by p53 and other transcriptional factors. In NSCLC, miR-145 can inhibit tumor cell growth and invasion by targeting key regulatory genes, such as c-Myc and mucin 1 [18,39,40]. Several studies have explored the association between circulating miR-145 expression levels and its potential clinical utility in lung cancer diagnosis, suggesting that miR-145 may serve as a promising biomarker for clinical application [41,42].

By investigating the expression patterns of the previously stated miRNAs in exosomal and non-exosomal forms (serum and tissue), we aimed to gain deeper insights into their potential use as diagnostic and prognostic biomarkers for lung carcinogenesis and evaluate which form could be more promising in terms of accuracy, sensitivity, and specificity. Our findings revealed distinct expression patterns between the different forms of miRNAs, suggesting a complex interplay during lung carcinogenesis. For the three investigated miRNAs, it was obvious that the expression of exosomal miRNAs dynamically changed during the induction process. 

Despite being more abundant, circulating cell-free miRNAs are susceptible to degradation by extracellular RNases. This susceptibility poses challenges to their quantification and reliability as biomarkers. Previous research has demonstrated that exosomal miRNAs exhibit greater stability in bodily fluids than their non-exosomal counterparts. In the study by Sanz-Rubio et al. [13], miRNAs isolated from exosomes exhibited greater long-term stability over time. This enhanced stability can be attributed mainly to the protective nature of the exosomal lipid bilayer. However, not all miRNAs are loaded in exosomes, and the miRNA content within exosomes could sometimes differ from the miRNA profile of their originating cells, which is known as the selective sorting of miRNAs [43]. The research on the mechanism through which specific miRNAs are selectively enclosed into exosomes is an active research area. The selective sorting of miRNAs into exosomes involves several proposed mechanisms, including specific sequence motifs within the miRNA sequence (EXO-motif), which influence their preferential loading into EVs through RNA-binding proteins like sumoylated hnRNPA1 [22,44]. Interestingly, the 3′ ends of the miRNA could also be crucial for sorting miRNA into exosomes. It was observed that miRNAs with adenylated 3′ ends are relatively enriched within cells, and on the other hand, the isoforms with uridylated 3′ ends are more abundant in exosomes, suggesting that post-transcriptional modifications (adenylation and uridylation) may contribute to sorting of miRNAs into exosomes [45]. Another regulation layer involves the effect of hormones on exosome production and selective sorting. For instance, 17β-estradiol was found to increase extracellular vesicle production and selectively package specific miRNAs, particularly the let-7 family, into these vesicles [44].

There have been few studies comparing EV-derived miRNAs and cell-free miRNAs. However, in these limited studies, it was evident that certain exosomal miRNAs showed higher accuracy in reflecting the physiological and pathological conditions of their originating cells compared to circulating cell-free miRNAs [14,34,46]. In an initial meta-analysis that included ten types of cancer, exosomal miR-21 showed greater accuracy for diagnosis compared to non-exosomal circulating miR-21 in various carcinomas, including lung cancer [34]. In the study by Endzelinš et al. [14], miR-200c-3p and miR-21-5p showed better diagnostic performance in differentiating between prostate cancer and benign prostatic hyperplasia patients when analyzed in plasma EVs (AUC = 0.68 and AUC = 0.67) compared to their expression levels in whole plasma (AUC = 0.48 and AUC = 0.47), respectively. However, for miR-375, better diagnostic performance was detected when tested in whole plasma compared to EVs (AUC = 0.68 vs. AUC = 0.64, respectively).

The performance of miRNA as a biomarker can be distinctive based on the source. In the study by Crossland et al. [46], miR-423, miR-199, and miR-93* from serum or EVs showed diagnostic potential for acute graft-versus-host disease (aGvHD). However, the serum forms of these miRNAs were associated more with aGvHD incidence and severity, whereas their EV-associated forms exhibited greater predictive power. These findings suggest the superiority of specific exosomal miRNAs for use as reliable biomarkers in liquid biopsy applications for predictive purposes.

These findings align with our results, suggesting that the diagnostic performance of miRNAs can vary depending on their source, with certain miRNAs exhibiting superior accuracy in exosomal versus non-exosomal compartments.

Exosomal proteins have great potential as diagnostic biomarkers in cancer. Previous studies have focused on identifying exosomal proteins that hold the potential to serve as diagnostic and prognostic biomarkers of lung cancer. Exosomal proteomes have identified several exosomal proteins, including CD317, EGFR, leucine-rich a2-glycoprotein (LRG1), alpha-2-HS-glycoprotein (AHSG), and extracellular matrix protein 1 (ECM1), that hold the potential to serve as indicators for lung cancer early detection [47].

Our study also assessed the diagnostic performance of clinically applied protein markers CEA and CYFRA 21-1 in exosomal and serum compartments. Although CEA and CYFRA 21-1 are considered promising serum tumor biomarkers for lung cancer diagnosis when combined or used individually [48], their exosomal form has not been investigated before in this context. According to our findings, both exosomal and serum CEA exhibited high diagnostic accuracy with an AUC of 0.960 and 0.944, suggesting the utility of CEA either in serum or exosomal form for lung cancer detection. However, exosomal CYFRA 21-1 showed a slightly lower AUC (0.880) compared to serum CYFRA 21-1 (AUC = 1.000), suggesting that while exosomal CYFRA 21-1 is valuable, serum levels may provide more reliable diagnostic information.

The results of our study suggest that integrating exosomal protein with other exosomal cargo, such as miRNAs, may lead to increased diagnostic accuracy compared to employing either indicator individually. This collaborative approach emphasizes the possibility of utilizing a group of exosomal biomarkers to enhance diagnostic accuracy.

While our analysis compared serum-derived exosomal miRNAs with free serum and tissue miRNA levels, isolating exosomes directly from tumor tissue could further clarify whether the same miRNA signatures observed in the bloodstream originate from cancer cells. A previous study found that tissue-derived EVs isolated from the interstitial space of tissues could accurately reflect the actual physiological or pathological state of the tissue microenvironment compared to biofluid EVs [49]. Therefore, future work should include the isolation of exosomes from resected lung tumors. Such an approach would help confirm the tumor specificity of these biomarkers and further strengthen their predictive power in early lung carcinogenesis.

Our study provides valuable insights into the sensitivity of exosomal miRNAs and proteins as diagnostic biomarkers for early diagnostic purposes and monitoring progress during lung carcinogenesis. However, it is crucial to emphasize that our investigation was conducted in a rat model, and more validation in human samples is required to assess the clinical impact of exosomal biomarkers. Moreover, there are differences between rat and human lung tumors. Replicating well-defined lung cancerous lesions has proven to be particularly challenging. Nevertheless, carcinogen-induced lung tumors in rats exhibit a degree of histopathological and molecular similarity to their human counterparts, supporting the translational value of this model [50].

Before recommending exosomal biomarkers for clinical use, it is important to consider the lack of standardized extraction and detection methods. This results in inconsistency among different study cohorts, even in analyzing the same exosomal miRNAs or proteins, thus limiting their widespread clinical application as promising biomarkers. Moreover, the isolation of exosomal miRNAs by the traditional methods is more technically challenging than isolating non-exosomal biomarkers, which creates additional challenges for routine clinical use. Traditional methods such as ultracentrifugation are often time-consuming, require large sample volumes, and are not easily scalable for high-throughput workflows. However, recent technological advances, particularly the development of microfluidic platforms, have successfully addressed these limitations by enabling rapid, label-free, and high-throughput isolation of exosomes directly from small volumes of biofluids [51,52,53]. These innovations significantly streamline the isolation process and hold promise for integration into point-of-care diagnostic testing. As these technologies continue to advance, they are expected to greatly improve the feasibility and accessibility of exosomal content as reliable clinical biomarkers.

## 4. Materials and Methods

### 4.1. Experimental Animals

The animal experiment was conducted in accordance with the ARRIVE guidelines for the appropriate execution of animal experiments, with the approval of the Ethical Committee of the National Research Centre (protocol code # 130601100—approval date 6 February 2023). All procedures were performed in accordance with the NRC institutional guidelines for the care and use of laboratory animals. The principles of the 3Rs (Replacement, Reduction, Refinement) were followed in an attempt to minimize animal suffering and decrease the number of animals utilized.

The experimental work involved pathogen-free male Sprague Dawley rats weighing 160 ± 20 g (estimated age, 6–8 weeks old). Animals were provided from the animal facility of the National Research Centre. Following a one-week acclimatization period, animals were housed in groups (n = 10/group) and kept at a temperature of 26 ± 2 °C, following a regular 12 h cycle of light and darkness. The rats were provided with commercially available pellet food and water ad libitum for a one-week acclimatization period.

### 4.2. Induction of Lung Carcinogenesis

**Figure 7:**
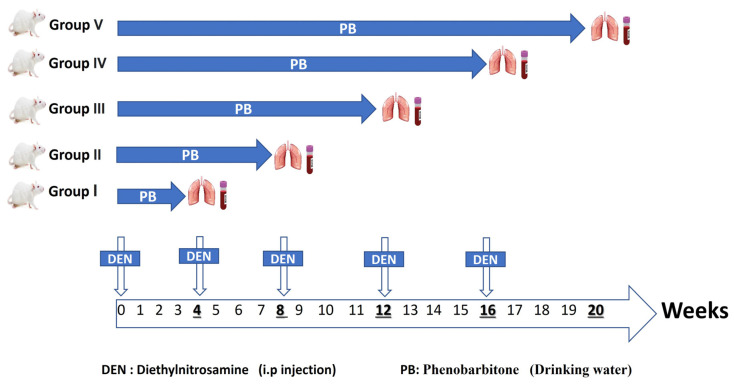
Schematic diagram illustrating the experimental timeline and design for the chemical-induced lung carcinogenesis model. The diagram shows the timing of five intraperitoneal DEN injections (150 mg/kg body weight) administered at weeks 0, 4, 8, 12, and 16. Simultaneously, treated animals received 0.05% PB in their drinking water continuously for 20 weeks. Sample collection points from the DEN/PB-treated groups are indicated at 4, 8, 12, 16, and 20 weeks, representing different durations of carcinogen exposure. A parallel control group received equivalent volume saline IP injections and regular drinking water, and was sampled at the end of the 20-week study.

A lung carcinogenesis model was conducted following a previously established protocol with modifications [54,55,56]. The study involved a total of 60 animals, allocated to six experimental groups. Following the acclimatization period, animals were randomly assigned to either the control group or the tumor-induced group. Group allocation was performed by the lead investigator, who assigned animals randomly to treatment and control groups. Personnel administering the treatments were aware of the group allocation. The study employed intraperitoneal injections of N-Diethylnitrosamine (DEN) at a dose of 150 mg/kg body weight in a solution of 0.9% NaCl. A total of five DEN injections were administered at 4-week intervals throughout the 20-week study period (on Day 0, and at the beginning of weeks 4, 8, 12, and 16). In parallel, the rats were administered a 0.05% phenobarbitone (PB) solution in their drinking water for the entire 20-week duration. To track tumor progression, samples were collected at five time points: 4, 8, 12, 16, and 20 weeks. Each time point represents a different duration of carcinogen exposure (Figure 7). The control group (n = 10) received IP injections of an equivalent volume of 0.9% NaCl vehicle at the same time points as the DEN injections and received regular drinking water ad libitum.

Stage I (4-week induction): Rats received one dose of DEN and 4 weeks of PB administration.

Stage II (8-week induction): Rats received two doses of DEN and 8 weeks of PB administration.

Stage III (12-week induction): Rats received three doses of DEN and 12 weeks of PB administration.

Stage IV (16-week induction): Rats received four doses of DEN and 16 weeks of PB administration.

Stage IV (20-week induction): Rats received five doses of DEN and 20 weeks of PB administration.

The animals were monitored regularly throughout the study. This monitoring included assessments of general appearance, activity level, body weight, food and water intake, and the presence of any clinical signs. At each specified time interval, an orbital sinus puncture was conducted to collect blood samples under brief anesthesia. Blood was allowed to clot at room temperature for 30 min, followed by centrifugation at 1600× *g* for 10 min at 4 °C to obtain serum. Serum was aliquoted into new microcentrifuge tubes and stored at −80 °C for subsequent exosome isolation and RNA extraction. Following blood collection, animals were euthanized. Lung tissue was carefully collected from all animals. Gross examination was performed to identify potentially cancerous lesions, and tissue was sampled accordingly. Lung tissue was then divided into two portions: one was immediately fixed in 10% neutral buffered formalin for histopathological analysis, and the other was snap-frozen in liquid nitrogen and stored at −80 °C for subsequent RNA extraction.

### 4.3. Histopathological Examination

Lung tissue samples were excised from rats at distinct time points corresponding to different stages of the carcinogenesis process. These tissue samples were embedded in paraffin blocks and sectioned into 5 μm-thick slices on slides. The tissue sections were subjected to the Hematoxylin and Eosin (H&E) staining protocol, followed by microscopic analysis to identify and characterize the histological abnormalities specific to each time point of the carcinogenesis process.

### 4.4. Exosome Isolation and Characterization

#### 4.4.1. Exosome Isolation

Serum samples were thawed on ice and then centrifuged at 3000× *g* for 10 min at 4 °C to remove cellular debris. The supernatant was then transferred to new tubes for exosome isolation. Exosomes were isolated from 500 µL of serum samples using the miRCURY Exosome Serum/Plasma Kit (Cat. No# 76603, Qiagen, Hilden, Germany) following the manufacturer’s instructions. The miRCURY Exosome Kits employ a precipitation approach for the isolation and enrichment of exosomes. Integrating the initial sample with the precipitation buffer reduces the hydration of the subcellular particles, enabling the precipitation of small EV particles through a low-speed centrifugation process. Briefly, 500 µL of serum samples were thawed on ice and gently mixed with 200 μL of precipitation buffer. The mixture was incubated for 60 min at 4 °C, then centrifuged at 1500× *g* for 30 min at 20 °C. The supernatant was completely discarded, and the pellet was centrifuged again to remove the residual supernatant. The pellet was dissolved in the exosome resuspension buffer provided in the kit, with gentle pipetting to avoid exosome disruption. To minimize the risk of RNase contamination, we proceeded directly with further downstream sample processing (RNA extraction and TEM characterization). Part of the resuspended exosome pellets was stored at −80 °C for protein analysis. Characterization techniques were conducted to identify the identity and purity of isolated exosomes through transmission electron microscopy (TEM), dynamic light scattering, and Western blotting for the exosomal marker CD63. The concentration of exosomal protein was measured by the BCA protein assay for normalization purposes.

#### 4.4.2. Transmission Electron Microscopy (TEM) and Dynamic Light Scattering (DLS)

The morphology and size distribution of the isolated exosomes were characterized for some samples to confirm the successful isolation of exosomes. High-resolution imaging techniques like transmission electron microscopy (TEM) are the primary techniques for assessing the morphology of isolated extracellular vesicles [57]. The freshly extracted exosome suspension was fixed in 2% paraformaldehyde for 1 h. After fixing, the exosome suspension was placed onto formvar–carbon-coated nickel grids and allowed to adsorb for 10–15 min before being gently removed with PBS. To negatively stain the exosomes, 2% uranyl acetate was applied to the grid for 30 s and then removed using the Whatman filter paper. The grids were thoroughly dried before the examination using the transmission electron microscopy HR-TEM, JEM-2100 (JEOL Ltd., Tokyo, Japan), operated at 160 kV. Images were captured using an attached Gatan CCD camera operated by Gatan Microscopy Suite version 2.11.1404.0 (Gatan Inc., Pleasanton, CA, USA). 

The size distribution, the average diameter, and the zeta potential of extracted exosomes were evaluated using a particle size analyzer (Nano-ZS, Malvern Instruments Ltd., Malvern, Worcestershire, UK). As described previously [58], the sample was first diluted with PBS, and then the hydrodynamic sizes of the purified exosomes were measured.

#### 4.4.3. Total Protein Content

Total protein contents of exosomes were measured as an indicator of the quality and integrity of isolated exosomes and for normalizing samples for the subsequent Western blot analysis. Lysis of exosomes was first performed with RIPA buffer with a protease inhibitor cocktail, and then the samples were sonicated for 5 min. Total protein content was measured using PierceTM BCA protein assay kit (cat# 23225; Thermo Scientific, Waltham, MA, USA) with a standard curve (range 0–2000 μg/mL) from bovine serum albumin (BSA).

#### 4.4.4. Western Blot

Equal amounts of exosomes lysed in RIPA buffer were mixed with 4x Laemmli sample buffer, including 2-mercaptoethanol, and then samples were denatured at 95 °C for 10 min.

Western blot was performed as described previously [59]. We used an anti-CD63 antibody (Elabscience^®^, Wuhan, China) (Cat. No# E-AB-53280) to detect the extracted exosomes. Normalized samples were electrophoresed in a 12% SDS-PAGE gel (TGX Stain-Free™ FastCast™ Acrylamide kit, Bio-Rad, Hercules, CA, USA). The gel was then transferred to a PVDF membrane (Trans-Blot Turbo RTA mini transfer kit, Bio-Rad, Hercules, CA, USA). PVDF membrane was then blocked with 5% BSA blocking buffer before overnight incubation with the anti-CD63 antibody (1:1000). The membrane was then washed three times in TBST buffer before incubation with the secondary antibody Anti-rabbit IgG, HRP-linked (Cat. No # 7074, Cell Signaling Technology^®^, Danvers, MA, USA), for 1 h. For protein bands visualization, the membrane was subjected to HRP chemiluminescent substrate (SuperSignal™ West Pico Chemiluminescent Substrate, Thermo Fisher Scientific, Waltham, MA, USA). The membrane was visualized using a ChemiDoc Imaging System for Image Acquisition (Bio-Rad, Hercules, CA, USA).

### 4.5. Selection of Candidate miRNAs

**Figure 8:**
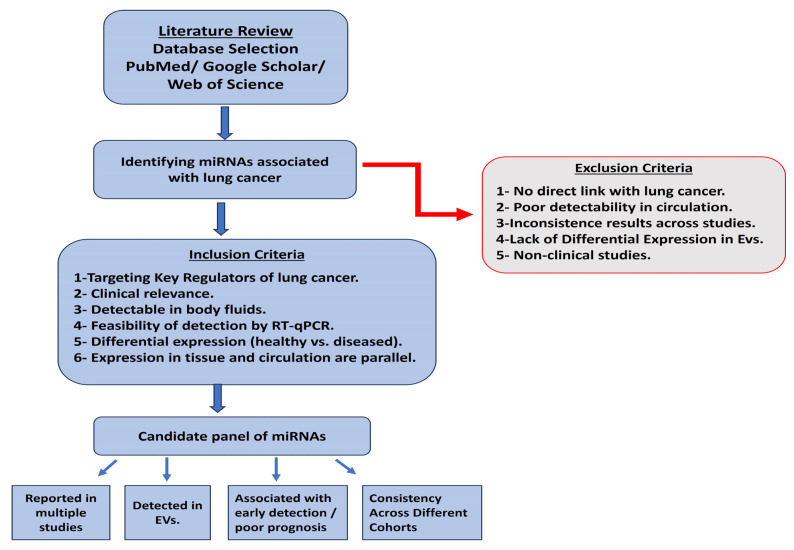
A flow chart of the miRNA selection process.

For our research, it was essential to carefully consider the relevance of our selected miRNAs to lung cancer development and their potential diagnostic and prognostic value. We conducted a thorough literature review of existing studies on lung cancer-associated miRNAs (Figure 8). Our inclusion criteria for the candidate miRNAs included the selection of miRNAs that directly target and control prominent key regulators of lung cancer development and progression, previously proven to be detectable in body fluids, exhibit significant differences between healthy and diseased individuals, and can be analyzed using RT-qPCR [15,20,23,60]. We also focused on studies utilizing EVs, specifically those profiling the differential expression of EV-miRNAs between lung cancer and healthy individuals [34,61,62]. Using our comprehensive selection process, we identified a list of candidate miRNAs highly relevant to lung cancer (Supplementary Table S1). From this list, we selected two oncogenic miRNAs (miR-21 and miR-19b) and one tumor suppressor miRNA (miR-145) for further evaluation.

### 4.6. RNA Extraction from Isolated Exosomes, Serum, and Tissue Samples and Expression Analysis

In our study, we extracted RNA from isolated serum-derived exosomes, serum samples, and corresponding lung tissue samples using suitable RNA extraction protocols for each. For exosomal RNA and serum RNA, we used the miRNeasy Serum/Plasma Kit (Cat. No#217184, Qiagen, Hilden, Germany).

For tissue RNA, lung tissue sections were first homogenized using a magnetic bead-based extraction method on the QIAGEN TissueLyser II system (Qiagen GmbH, Hannover, Germany), and then total RNA was extracted using the miRNeasy RNA isolation kit (Cat. No# 217004, Qiagen, Hilden, Germany) following the Qiagen supplementary protocol. The quality and quantity of extracted RNAs were evaluated spectrophotometrically using Nanodrop-1000.

Reverse transcription was conducted to convert RNA into cDNA using miRCURY LNA Reverse Transcription Kit (Qiagen, Cat#339340) following the provided guidelines. A fixed concentration of total RNA was reverse transcribed. Next, we conducted RT-qPCR using the miRCURY LNA SYBR Green PCR Kit (Cat#339346). Primers were selected from miRCURY LNA miRNA PCR assays for miR-21-5p, miR-19b-3p, miR-145-5p, miR-16, and U6 snRNA. The cycling condition was set as follows: initial heat activation for 2 min at 95 °C, followed by 40 cycles of a two-step cycling of denaturation for 10 s at 95 °C, and a combined annealing/extension step at 56 °C for 60 s. To analyze the expression of exosomal and serum miRNAs, the target miRNAs were normalized to miR-16. For tissue miRNAs, the expression was normalized to U6 snRNA. All RT-qPCR experiments were conducted on the QuantStudio™5 Real-Time PCR System (Applied Biosystems, Thermo Fisher Scientific, Waltham, MA, USA). The relative expression of interest was normalized and calculated using the 2^−ΔΔCt^ method.

### 4.7. Evaluating Levels of Serum and Exosomal Carcinoembryonic Antigen (CEA) and Cytokeratin Fragment Antigen-21 (CYFRA 21-1)

The levels of Carcinoembryonic Antigen (CEA) and Cytokeratin Fragment Antigen-21 (CYFRA 21-1) were quantified in serum and exosomal lysates using enzyme-linked immunosorbent assay (ELISA) kits following the manufacturer’s instructions. Briefly, serum samples and exosomal lysates were diluted appropriately and added to pre-coated ELISA plates specific for CEA (SunLong Biotech Co., Ltd., Hangzhou, China, Cat. No# SL1244Ra) and CYFRA 21-1 (SunLong Biotech Co., Ltd, Cat. No#L1740Ra). After incubation and washing, enzyme-linked antibodies were added to the plates to detect the bound antigens. Colorimetric reactions were developed using substrate solutions, and absorbance was measured at specific wavelengths using a microplate reader. The optical density (OD) values acquired were converted into concentrations (ng/mL) of CEA and CYFRA 21-1 using standard curves developed with known concentrations of the corresponding recombinant proteins included in the kits. The measurements of serum and exosomal CEA and CYFRA 21-1 levels were performed in duplicates for each sample to ensure accuracy and reproducibility.

### 4.8. Statistical Analysis

Quantitative data were statistically represented in terms of mean ± standard deviation (SD). The present study compared different groups using one-way ANOVA followed by Tukey’s Honestly Significant Difference (HSD) test for multiple comparisons. The receiver operating characteristic (ROC) curve analysis was conducted to generate the cut-off value, the area under the curve (AUC), and the sensitivity and specificity for the parameters in the present study. All statistical analysis were performed using the statistical software SPSS (Statistical Package for Social Science) program version 21.0. Graphs were sketched using GraphPad Prism version 8.0.1 and the MedCalc program version 22.0.

## 5. Conclusions

In conclusion, our findings demonstrate distinct expression patterns of the selected miRNAs within exosomes compared to their non-exosomal counterparts during lung cancer development. Exosomal miR-19b emerged as a promising candidate for early detection due to its robust and consistent upregulation during the induction process and its strong correlation with the corresponding serum and tissue forms.

Future research should focus on verifying these biomarkers in larger, more diverse patient cohorts to assess their clinical value and investigate the underlying mechanisms driving their differential expression.

## Figures and Tables

**Table 1:** Diagnostic performance of selected miRNAs and protein tumor biomarkers from exosomal and non-exosomal sources in differentiating early-stage lung carcinogenesis from controls.

Parameters	AUC	Cut-Off Value	Sensitivity %	Specificity %	*p* Value	95% CI
Exo-miR-19b	0.920	1.294	80.0%	100.0%	0.028	0.738–1.000
Serum miR-19b	1.000	1.215	100.0%	100.0%	0.009	1.000–1.000
Tissue miR-19b	0.880	1.170	80.0%	100.0%	0.047	0.640–1.000
Exo-miR-21	1.000	1.279	100.0%	100.0%	0.009	1.000–1.000
Serum miR-21	0.625	1.018	66.7%	66.7%	0.471	0.288–0.962
Tissue miR-21	0.880	1.235	80.0%	80.0%	0.047	0.662–1.000
Exo-miR-145	1.000	0.816	100.0%	100.0%	0.009	1.000–1.000
Serum miR-145	1.000	0.663	100.0%	100.0%	0.004	1.000–1.000
Tissue miR-145	0.520	1.058	60.0%	60.0%	0.917	0.135–0.905
Exo-CEA	0.960	4.413	80.0%	100.0%	0.016	0.843–1.000
Serum CEA	0.944	4.750	83.3%	100.0%	0.010	0.814–1.000
Exo-CYFRA21	0.880	2.688	80.0%	100.0%	0.047	0.640–1.000
Serum CYFRA21	1.000	1.719	100.0%	100.0%	0.009	1.000–1.000

**Table 2:** Spearman’s correlation coefficient for miRNAs and protein tumor biomarkers across exosomal, serum, and tissue samples. * Correlation is significant at *p* < 0.05. ** Correlation is significant at *p* < 0.01.

	r (Correlation Coefficient)	*p* Value
Exo-miR-19b with serum miR-19b	0.772 **	*p* < 0.001
Exo-miR-19b with tissue miR-19b	0.718 **	*p* < 0.001
Exo-miR-21 with serum miR-21	0.533 **	*p* < 0.002
Exo-miR-21 with tissue miR-21	0.699 **	*p* < 0.001
Exo-miR-145 with serum miR-145	0.699 **	*p* < 0.001
Exo-miR-145 with tissue miR-145	0.656 **	*p* < 0.001
Exo-CEA with serum CEA	0.658 **	*P* < 0.001
Exo-CYFRA21 with serum CYFRA21	0.585 **	*p* = 0.001
Exo-CEA with Exo-CYFRA21	0.568 **	*p* = 0.001
Serum CEA with serum CYFRA21	0.745 **	*p* < 0.001
Exo-miR-19b with Exo-miR-21	0.676 **	*p* < 0.001
Exo-miR-19b with Exo-miR-145	−0.790 **	*p* < 0.001
Exo-miR-21 with Exo-miR-145	−0.622 **	*p* < 0.001
Exo-miR-19b with Exo-CEA	0.486 **	*p* = 0.007
Exo-miR-19b with Exo-CYFRA21	0.527 **	*p* = 0.003
Exo-miR-21 with Exo-CEA	0.361	*p* = 0.05
Exo-miR-21with Exo-CYFRA21	0.396 *	*p* = 0.03
Exo miR-145 with Exo-CEA	−0.565 **	*p* = 0.001
Exo miR-145 with Exo-CYFRA21	−0.668 **	*p* < 0.001

## Data Availability

The original contributions presented in this study are included in the article/Supplementary Materials. Further inquiries can be directed to the corresponding author upon reasonable request and with permission of the National Research Centre.

## Supplementary Materials

The following supporting information can be downloaded at https://www.mdpi.com/article/10.3390/ncrna11030047/s1, Figure S1: Receiver operating characteristic curves of diagnostic performance for miR-19b, miR-21, miR-145, CEA, and CYFRA21 in different sample types. Table S1: A short list of candidate miRNAs.

## Abbreviations

The following abbreviations are used in this manuscript:

Ago	Argonaute
AHSG	Alpha-2-HS-Glycoprotein
aGvHD	Acute Graft-versus-Host Disease
CEA	Carcinoembryonic Antigen
CYFRA 21-1	Cytokeratin Fragment Antigen-21
DEN	N-Diethylnitrosamine
DLS	Dynamic Light Scattering
ELISA	Enzyme-linked Immunosorbent Assay
EV	Extracellular Vesicle
HDL	High-density lipoproteins
LRG1	Leucine-Rich a2-Glycoprotein
NSCLC	Non-Small Cell Lung Cancer
PB	Phenobarbitone
PTEN	Phosphatase and Tensin Homolog
TEM	Transmission Electron Microscopy
