# Supplementary material for: Diagnostic Potential of Exosomal and Non-Exosomal Biomarkers in Lung Cancer: A Comparative Analysis Using a Rat Model of Lung Carcinogenesis
Source: Noncoding RNA. 2025 Jun 16;11(3):47. doi: 10.3390/ncrna11030047 (PMC12196065; doi:10.3390/ncrna11030047)
Supplement: Supplementary file 1 [file ncrna-11-00047-s001.zip › Supplementary Figure 1.pdf]

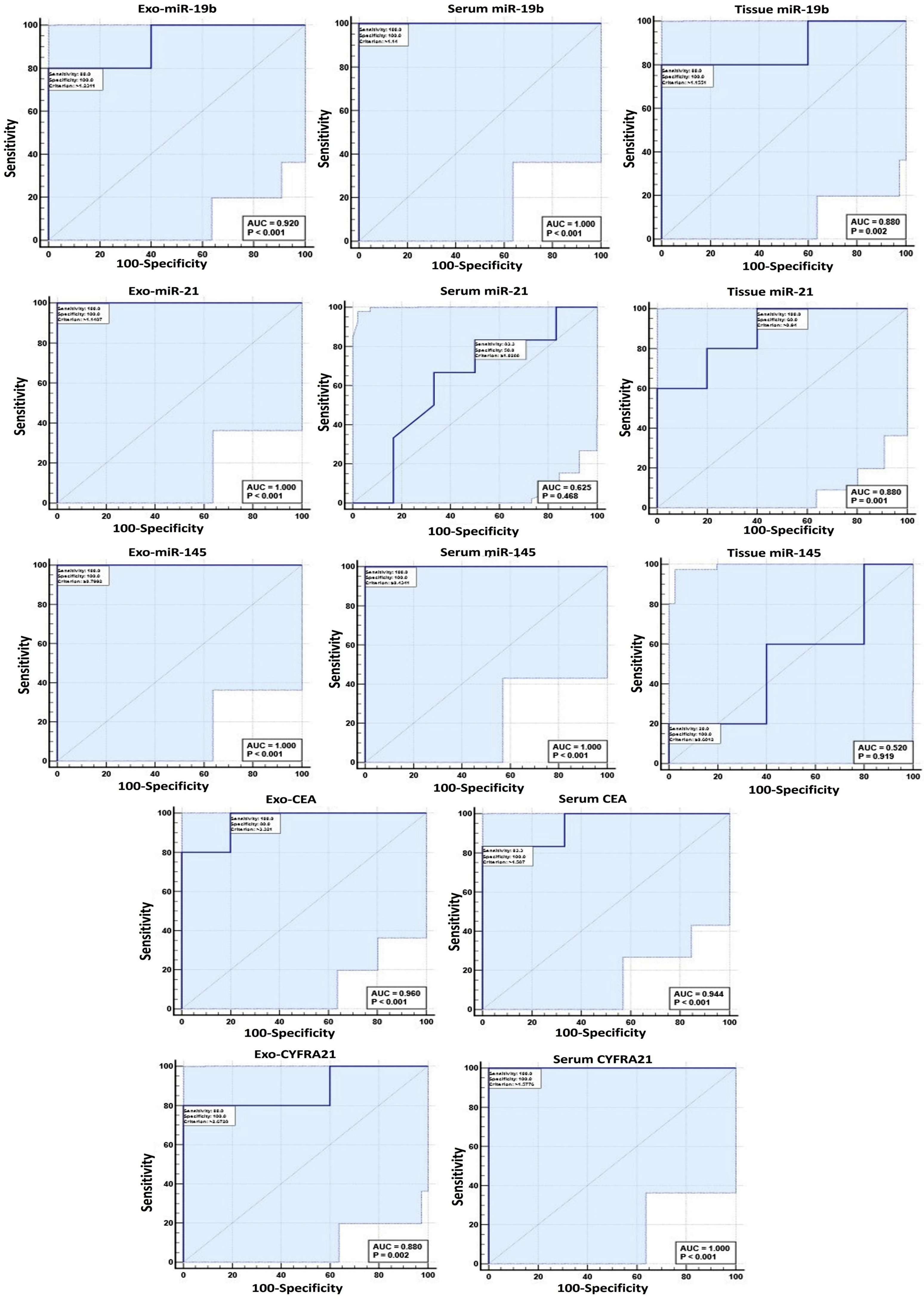

Exo-miR-19b
Sensitivity
100-Specificity
AUC = 0.920
P < 0.001
Serum miR-19b
AUC = 1.000
P < 0.001
Tissue miR-19b
AUC = 0.880
P = 0.002
Exo-miR-21
AUC = 1.000
P < 0.001
Serum miR-21
AUC = 0.625
P = 0.468
Tissue miR-21
AUC = 0.880
P = 0.001
Exo-miR-145
AUC = 1.000
P < 0.001
Serum miR-145
AUC = 1.000
P < 0.001
Tissue miR-145
AUC = 0.520
P = 0.919
Exo-CEA
AUC = 0.960
P < 0.001
Serum CEA
AUC = 0.944
P < 0.001
Exo-CYFRA21
AUC = 0.880
P = 0.002
Serum CYFRA21
AUC = 1.000
P < 0.001
