# Supplementary material for: Diagnostic Potential of Exosomal and Non-Exosomal Biomarkers in Lung Cancer: A Comparative Analysis Using a Rat Model of Lung Carcinogenesis
Source: Noncoding RNA. 2025 Jun 16;11(3):47. doi: 10.3390/ncrna11030047 (PMC12196065; doi:10.3390/ncrna11030047)
Supplement: Supplementary file 1 [file ncrna-11-00047-s001.zip › Supplementary table 1.pdf]

Supplementary table S1: A short list of candidate miRNAs

miRNA	Function	Sample Type
**miR-21-5p**	Oncogenic	Serum/plasma/EVs
**miR-19b-3p**	Oncogenic	Serum/plasma/EVs
**miR-145-5p**	Tumor suppressor	Serum/plasma/EVs
**miR-19a-3p**	Oncogenic	Plasma
**miR-10b-5p**	Oncogenic	Plasma exosomes
**miR-155-5p**	Oncogenic	Serum/plasma/EVs
**let-7d-5p**	Tumor suppressor	Serum
**let-7e-5p**	Tumor suppressor	Serum
**miR-15a-5p**	Tumor suppressor	Serum
**miR-25-3p**	Tumor suppressor	Serum
**miR-148a-3p**	Tumor suppressor	Serum
**miR-192-5p**	Tumor suppressor	Serum
**miR-126-3p**	Tumor suppressor	Serum/exosomes
**miR-23b-3p**	Oncogenic	Plasma-derived EVs
**miR-10b-5p**	Oncogenic	Plasma-derived EVs
**miR-17-5p**	Oncogenic	Plasma
**miR-574-5p**	Oncogenic	Plasma-derived EVs
**miR-181a-5p**	Oncogenic	Plasma-derived EVs
**miR-210**	Oncogenic	Serum
**miR-126**	Tumor suppressor	Serum/exosomes
